# Nursing strikes among infants and its affecting factors in Rafsanjan city

**DOI:** 10.25122/jml-2020-0118

**Published:** 2021

**Authors:** Fatemeh Jalali, Zahra Kamiab, Morteza Khademalhosseini, Fatemeh Daeizadeh, Gholamreza Bazmandegan

**Affiliations:** 1. Clinical Research Development Unit, Ali-Ibn Abi-Talib Hospital, Rafsanjan University of Medical Sciences, Rafsanjan, Iran; 2. Department of Pediatrics, Ali-Ibn Abi-Talib Hospital, School of Medicine, Rafsanjan University of Medical Sciences, Rafsanjan, Iran; 3. Department of Family Medicine, Ali-Ibn Abi-Talib Hospital, School of Medicine, Rafsanjan University of Medical Sciences, Rafsanjan, Iran; 4. Department of Pathology, Ali-Ibn Abi-Talib Hospital, School of Medicine, Rafsanjan University of Medical Sciences, Rafsanjan, Iran

**Keywords:** nursing strike, infants, affecting factors, playfulness, refusal to breastfeed

## Abstract

The infant’s refusal to breastfeed can be a stressful and concerning matter for a mother. This study aimed to investigate the frequency and factors leading to nursing strikes in Rafsanjan city. This descriptive study was performed on infants who had been referred to the pediatrician’s office with a complaint of a nursing strike. The research sample included 70 infants, and all the required data, including the causes of the nursing strike and the demographic information of the mother and the infant, were collected using a checklist. The Statistical Package for the Social Sciences (SPSS) software version 20 was used to analyze the data. The percentage was used to express qualitative indices, and the mean and standard deviation were used to express quantitative indices. The results showed that the most common factors contributing to the infants’ breast refusal were playfulness and distraction (50%) and recent vaccinations in the last 12 days (48.6%). Besides, the most common maternal factors affecting breast refusal were level of education (67.1%), recent acute stress (41.4%), and inadequate milk production (35.7%). The results of the present study showed that playfulness and distraction of the baby, recent vaccination, use of a pacifier, level of education and recent stress of the mother, breastfeeding program, and insufficient milk production are the most common reasons for nursing strikes.

## Introduction

Breast milk is the best food that provides energy and most of the nutrients a child needs [1, 2]. Given that infancy is one of the most important and sensitive periods of life that requires special care and considering that breastfeeding can meet all the nutritional needs of the infant from birth to the end of six months, the World Health Organization and the American Academy of Pediatrics have recommended exclusive breastfeeding up to about 6 months after birth. “Exclusive breastfeeding” is defined as giving no other food or drink, not even water, except breast milk, as it provides all the vitamins and micronutrients needed by the infant. Recent research has shown that rapid initiation of breastfeeding can reduce the neonatal mortality rate by up to 22% [3]. Studies conducted in industrialized countries have shown that infants who are not breastfed for at least 6 months are 3.5 times more likely than other infants to be hospitalized for respiratory infections such as pneumonia or asthma [4–6].

The infant’s abrupt refusal to breastfeed is often called a “nursing strike” [7]. The nursing strike can be a stressful and concerning matter for a mother, and she might take this as a personal matter and believe her child is refusing her and not the breast milk. She might also think that something is missing in her milk or the milk is not insufficient for her child. In fact, a common reason for the cessation of nursing is the infant’s refusal to breastfeed. However, this problem can often be overcome. Having knowledge and focusing on this problem can prolong breastfeeding. The mother should be reassured that there is definitely a reason behind the infant’s refusal behavior, and by acknowledging it, she can encourage her newborn to re-breastfeed. It should be noted that most of the time, there is no specific reason for refusing to be breastfed. There is a need to look more closely at the possible causes of this problem to help the mother solve this difficulty and restart the mother-infant connection [8].

Breastfeeding strikes can occur at any age, in any group, and at any time during infancy for different reasons [7], which can be classified into three categories:

1. Infant-related factors: nasal obstruction, teething, reflux [1], vaccination pain, brain injuries, mother-infant separation [8], distraction and playfulness [9], oral thrush infection, ear infections, nasal congestion, oral herpes simplex infections, facial injuries [10], prematurity, colic, allergies, cleft palate, tongue nodules, oral diseases [11], and starting bottle-feeding [12];
2. Mother-related factors: stress, change of soap or perfume, change of body odor, change of diet, menstrual cycle changes [1], mastitis, change in the nursing pattern, drugs, flat nipples, breast congestion [8], herpes zoster infection in the fourth thoracic dermatome [13], mother-infant separation, and having a new nurse [14];
3. Milk production-related factors: slow or insufficient milk production [8].

There is no significant association between the nursing strike and gestational age, type of delivery, and underlying maternal diseases. At the same time, there is a significant relationship between the nursing strike and maternal education and work. Besides, “breastfeeding refusal” is more commonly experienced by working mothers or those with higher education. In fact, higher education of the mother increases the probability of employment of the mother and further mother-infant separation [8].

Given that more research is needed on infants’ nursing strikes, as well as the established benefits of breastfeeding in preventing malnutrition, especially in developing countries, and considering that the infant’s refusal to breastfeed is solvable, this study aims to evaluate the most probable causes of the nursing strike in infants admitted to the pediatrician’s office in Rafsanjan city.

## Material and Methods

In this descriptive study, after obtaining approval from the Ethics Committee, 70 infants with a nursing strike were entered the study by convenience sampling method in the first six months of 2018 and were examined by a pediatrician. All the required data, including the causes of the nursing strike and the demographic information of the mother and the infant, were collected using a checklist. The inclusion criteria were met by all infants who had nursing strikes, and their mothers had completed a consent form stating their full awareness of entering the study. The exclusion criteria covered the infants who had not been breastfed since birth, including the infants with congenital malformations, a history of underlying maternal diseases that had impaired infant nutrition from birth, and severe mental illness affecting the mother, such as schizophrenia. Information on infant related factors (playfulness and distraction, nasal obstruction, tooth growth, oral candidacies, vaccinations, use of a pacifier, and simultaneous feeding with formula) and mother-related factors including underlying diseases, employment, mastitis, breast congestion, change perfume or soap, recent acute stress, mother-infant separation, history of new drug use, re-pregnancy, a special diet, and insufficient milk production were collected by face-to-face interviews.

The data collected by the checklists then entered the Statistical Package for the Social Sciences (SPSS) software, version 20. The Kolmogorov-Smirnov test was used to test the normality of data distribution. The quantitative data results were reported as mean and standard deviation, and the qualitative data results as a percentage. The Chi-square test or Fisher’s exact test was used to determine the frequency and causes of the nursing strike according to the independent and contextual variables. All statistical tests were performed at the significance level of 0.05.

## Results

**Figure 1.**
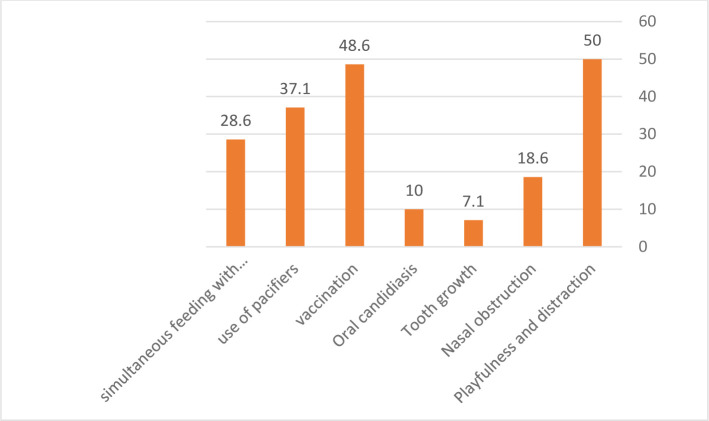
The frequency of infant-related factors accounting for nursing strikes.

The mean age of the infants in this study was 2.48±4.57 with a minimum of 1 and a maximum of 18 months. The age of most infants was between 3 and 4 months old (25.7%). Of these infants, 34 (48.6%) were boys, and 36 (51.4%) were girls. The mean age of mothers in this study was 27.65±4.89, and sixty-six of them (94.3%) were between 18 and 35 years old. Also, the level of education of most mothers of these infants (67.1 %) was diploma and less. Figure 1 shows the frequency distribution of infant-related factors. As can be seen, playfulness and distraction (50%), recent vaccinations in the last 12 days (48.6%), and the use of pacifiers (37.1%) are the most common causes of the nursing strike, respectively.

**Figure 2.**
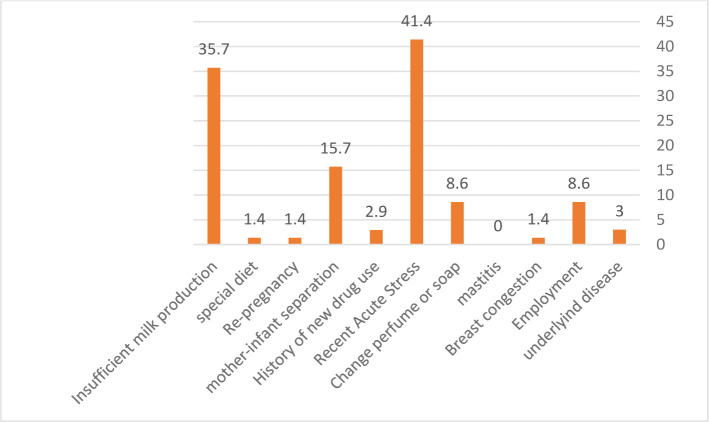
The frequency of mother-related factors accounting for nursing strikes.

Figure 2 shows the frequency of mother-related factors accounting for the nursing strike. As can be seen, the recent acute stress (41.4%) and insufficient milk production (35.7%) are the most common mother-related factors accounting for the infants’ nursing strike. The relationship between maternal causes and age in the Chi-square test showed that 100% of mothers who use formula milk and pacifiers are over 35 years old (Fisher’s exact test was 0.005 and 0.016, respectively).

## Discussion

In this study, 70 infants were admitted to infants’ offices in Rafsanjan with a complaint of nursing strikes in the first six months of 2018, and the infants were examined by a pediatrician. The results showed that playfulness and distraction (50%), recent vaccinations (48.6%), and the use of pacifiers (37.1%) were the most common infant-related factors contributing to the nursing strike. Besides, low maternal education (67.1%), recent acute stress (41.4%), inadequate milk production (35.7%), and the breastfeeding schedule (21.4%), respectively, were found as the most common mother-related factors accounting for the nursing strike.

It was shown that nutrition has the most significant effect on infant growth and development compared to other factors. It is clear that breast milk is the most appropriate source of nutrition for an infant. Experts believe that after giving birth, no moment should be wasted to start breastfeeding. Immediately after birth, for several days afterward, a thick and yellow fluid called colostrum is secreted from the mother’s breast, which is rich in immunogenic substances for the infant [15]. The most important benefit of breastfeeding for infants is protection against gastrointestinal problems, respiratory diseases, and ear infections [16], protection against allergies [17], strengthening the intelligence of the infant [18], prevention of obesity when older [19], protection against childhood leukemia [20], prevention of type 1 diabetes [16], protection of premature infants against infection and hypertension at older ages, and a possible reduction in the risk of sudden infant death syndrome (SIDS) [21].

In a study by Nayyeri *et al.* on 175 infants with an average age of 5.28 months, 41 infants (24%) had nursing strikes, and the most common causes of the nursing strikes were flat nipples (14.3%) and breast congestion (11.9%). The most common factors affecting breast refusal were playfulness and distraction (50%), nasal obstruction (31%), vaccination pain (19%), labor injuries (9.5%), and tooth growth (4.8%) [5]. Similar to the present study, playfulness and distraction of the infant and receiving a recent vaccine were common causes of the nursing strike. There was a significant relationship between the nursing strike and maternal education and work, as indicated by a higher rate of “breastfeeding refusal” among the working mothers or those with higher education. In fact, higher education of the mother increases the probability of employment of the mother and further mother-infant separation [8].

Poorahmad-Garbandi *et al.* conducted a study entitled “Reasons for Termination of Breastfeeding among Women Referred to Bandar-Abbas Health Centers” and examined 100 women with infants under one year of age who had stopped breastfeeding. The most important causes of the cessation of breastfeeding among the mothers were lactation insufficiency (38%), maternal employment (20%), breast problems (7%), pregnancy (6%), maternal illness (5%), and the recommendation of the physician and health care personnel (5%) [22]. Besides, the most important causes of the cessation of breastfeeding related to the infants were child crying and restlessness (13%) and child disease (8%) [22].

In a study by Khayyati *et al.* entitled “Reasons for Terminating Breastfeeding before the Age of Two”, the mothers of 288 children were examined. The results showed that 58 infants had stopped breastfeeding before the age of 22 months. In five cases, the cause was unknown. The most important maternal factors accounting for the cessation of breastfeeding were lack of milk in mother’s breasts (18.9%), mother’s belief in the sufficiency of breastfeeding (18.9%), maternal illness (11.3%), pregnancy (7.5%), maternal employment (5.7%), breast problems (3.8%) and family issues (3.8%) [9]. The infant-related factors included the infant’s refusal to breastfeed for unknown reasons (17%), congenital diseases (5.7%), starting bottle-feeding (5.7%), and early supplementary feeding (1.8%) [12]. The reasons for breast refusal in the reviewed studies are the same as those reported in the present study.

In a study by Winchell entitled “Nursing Strike: Misunderstood Feelings”, the possible causes of the nursing strike were placed into two categories: maternal-related causes (changes in the mother’s body odor, diet, and the onset of maternal menstruation) and infant-related causes (oral thrush, ear infections, nasal congestion, tooth growth, and herpes simplex infections) [10]. The results of the mentioned studies are similar to the findings of the present study in terms of the reasons for refusing milk. 

Dalili *et al.* examined “Frequency of Exclusive Breastfeeding and its Affecting Factors in Tehran” among 154 infants and found that only 48 infants (31%) had exclusive breastfeeding, and the main reasons for mothers to stop breastfeeding before the age of two were the mother’s lack of interest in breastfeeding (25.93%) and insufficient milk (20.37%) [23].

In a study by Ruowei *et al.* entitled “Why do mothers stop breastfeeding?”, 1,323 infants and their mothers were examined. The mothers of 320 infants under one-month-old, 302 infants at the age of one to two months, 268 infants at the age of three to five months, 183 infants at the age of six to eight months, and 250 infants over nine months of age had stopped breastfeeding, and 45.5% of them cited the lack of adequate milk as the main reason for stopping breastfeeding [24]. This finding is in line with the present study that reported insufficient milk production as one of the reasons for stopping breastfeeding.

## Conclusion

The results of the present study showed that playfulness and distraction, recent vaccinations, use of a pacifier, the mother’s education level and recent stress, the breastfeeding schedule, and inadequate milk production were the most common causes of breast refusal. Our findings are useful for helping mothers overcome breastfeeding barriers and for health officials who are trying to create targeted interventions for breastfeeding.

## Acknowledgments

This article was extracted from Dr. Fatemeh Daeizadeh's dissertation for obtaining a Doctor of Medicine (MD) degree in general medicine from the Rafsanjan University of Medical Sciences. We would like to express our gratitude for their cooperation and assistance. The authors would also like to thank the Clinical Research Development Unit for its support and collaboration in the Ali-Ibn Abi-Talib Hospital, Rafsanjan University of Medical Sciences, Rafsanjan, Iran. 

### Ethical approval

The approval for this study was obtained from the Ethics Committee of the Rafsanjan University of Medical Sciences (Approval ID: IR.RUMS.REC.1397.031).

### Consent to participate

Informed written consent was collected from the mothers after explaining the study’s purpose and methods.

### Conflict of interest

The authors declare that there is no conflict of interest.
